# Extracellular vesicles derived from umbilical cord mesenchymal stromal cells show enhanced anti-inflammatory properties via upregulation of miRNAs after pro-inflammatory priming

**DOI:** 10.1007/s12015-023-10586-2

**Published:** 2023-07-20

**Authors:** Mairead Hyland, Claire Mennan, Rebecca Davies, Emma Wilson, Daniel P. Tonge, Aled Clayton, Oksana Kehoe

**Affiliations:** 1 Centre for Regenerative Medicine Research, School of Medicine at the RJAH Orthopaedic Hospital, Keele University, Oswestry, SY10 7AG UK; 2 Centre for Regenerative Medicine Research, School of Pharmacy and Bioengineering at the RJAH Orthopaedic Hospital, Oswestry, SY10 7AG UK; 3 Chester Medical School, University of Chester, Chester, CH2 1BR UK; 4 School of Life Sciences, Keele University, Keele, ST5 5BG UK; 5 Tissue Microenvironment Group, Division of Cancer and Genetics, School of Medicine, Cardiff University, Cardiff, CF14 4XN UK

**Keywords:** Umbilical cord mesenchymal stromal cell, Extracellular vesicles, Pro-inflammatory priming, Culture conditions, Immunomodulation

## Abstract

Autoimmune conditions, such as rheumatoid arthritis, are characterised by a loss of immune tolerance, whereby the immune cells attack self-antigens causing pain and inflammation. These conditions can be brought into remission using pharmaceutical treatments, but often have adverse side effects and some patients do not respond favourably to them. Human umbilical cord mesenchymal stromal cells (UCMSCs) present a promising alternative therapeutic due to their innate anti-inflammatory properties which can be strengthened using pro-inflammatory conditions. Their therapeutic mechanism of action has been attributed to paracrine signalling, by which nanosized acellular particles called ‘extracellular vesicles’ (EVs) are one of the essential components. Therefore, this research analysed the anti-inflammatory properties of UCMSC-EVs ‘primed’ with pro-inflammatory cytokines and at baseline with no inflammatory cytokines (control). Both control and primed EVs were co-cultured with un-pooled peripheral blood mononuclear cells (PBMCs; *n* = 6) from healthy donors. Neither control nor primed EVs exerted a pro-inflammatory effect on PBMCs. Instead, the primed EVs showed the immunosuppressive potential by increasing the expression of the anti-inflammatory protein FoxP3 in PBMCs. This may be attributed to the upregulated miRNAs identified in primed EVs in comparison to control EVs (miR-139-5p, miR-140-5p, miR-214-5p). These findings aid in understanding how UCMSC-EVs mediate immunosuppression and support their potential use in treating autoimmune conditions.

**Graphical Abstract:**

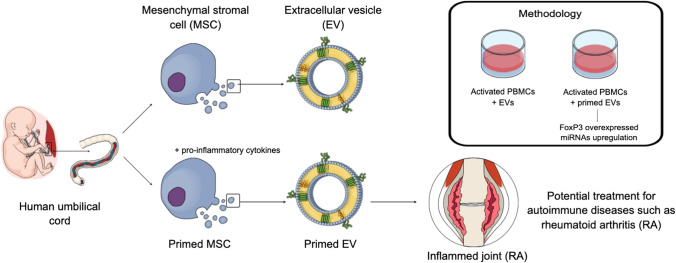

**Supplementary Information:**

The online version contains supplementary material available at 10.1007/s12015-023-10586-2.

## Introduction

Approximately, 4 million people in the UK live with an autoimmune disease [1]. This is defined by a breakdown in immune tolerance whereby the immune system targets self-antigens [2]. This leads to an imbalance of immune cells. Here, pro-inflammatory immune cells (Th1 and Th17 cells) are abundant over anti-inflammatory immune cells (Th2 and Treg cells) [3, 4]. Current pharmaceutical treatments aim to suppress immune responses but these treatments come with severe adverse side effects, therefore there is a need to develop alternative therapies to bring immune responses under control [5, 6]. The use of mesenchymal stromal cells (MSCs) as immune suppressors is one forerunner as multiple studies using in vitro, animal, and clinical studies show that MSCs can suppress Th1 and Th17 cells, promote Tregs, and restore the Th1/Th2 balance [7–10]. Our group has shown that when MSCs are injected intra-articularly into the joints of arthritic mice, they are effective in reducing knee swelling and TNF-α levels [11]. The therapeutic benefit of MSCs is largely attributed to their release of soluble factors and extracellular vesicles [12]. Previous research from our group showed that the conditioned media alone from MSCs was successful in reducing cartilage loss in mice with inflammatory arthritis, that which was associated with the increased presence of anti-inflammatory markers FoxP3 and IL-4 in the CD4 + T-cells [13].

MSC-mediated immunosuppression can be further enhanced when the MSCs are primed with pro-inflammatory cytokines [14, 15]. The practice of pro-inflammatory priming MSCs is common in regenerative medicine as it mimics the inflammatory in vivo environment and ‘activates’ MSCs [14]. Similarly activating MSCs before EV generation is likely to be effective in promoting a potentially therapeutic ‘anti-inflammatory’ EV, so this study explores both primed and control UCMSCs and UCMSC-EVs. We have previously demonstrated that pro-inflammatory primed EVs have an increased expression of proteins associated with migration and chemotaxis [16] and that pro-inflammatory primed EVs isolated from human bone marrow aspirates improved histopathological outcomes in inflammatory arthritis [17]. This research will focus on the functional properties of UCMSCs and their EVs, specifically if they can polarise PBMCs towards a more anti-inflammatory phenotype. In association with this, RNA sequencing was carried out to identify differentially expressed miRNAs between primed and control EVs since miRNAs contained within EV cargo are known to target multiple pathways, some of which govern these anti-inflammatory effects [18].

## Materials & Methods

### Isolation of MSCs from umbilical cord

Umbilical cords (*n* = 4) were collected from the Robert Jones and Agnes Hunt Orthopaedic Hospital (RJAH) following natural delivery with ethical approval (National Research Ethics Service; 10/H10130/62). Patient demographic information is shown in Table 1. The isolation of MSCs from the umbilical cord is described in Mennan et al. [19]. UCMSCs were first cultured on tissue culture plastic and then culture expanded in a Quantum® Cell Expansion System (Terumo BCT, Surrey, UK), as described in Mennan et al. [20].

**Table 1:** Demographic profile of the four umbilical cord donors

UCMSC	Age	Gender of newborn
Donor 1	35	Male
Donor 2	23	Male
Donor 3	24	Female
Donor 4	28	Male

Expansion of UCMSCs and collection of EV conditioned media.

UCMSCs were then grown up to passage 8. Media was changed every 2–3 days and contained DMEM F12, 10% Fetal Bovine Serum (FBS), 1% Penicillin–Streptomycin (P/S) (Life Technologies, Warrington, UK) for expansion. Upon achieving 80% confluence, UCMSCs were washed three times with PBS and the media changed to a composition of DMEM F12, 10% EV depleted FBS, 1% P/S for 48 h.

To deplete FBS of EVs, neat FBS was subject to ultracentrifugation at 120,000 g for 18 h at 4 °C using a Hitachi Himac Micro Ultracentrifuge CS150NX (Koki Holdings Co., Japan) [21, 22]. The FBS supernatant was then syringe filtered using a 0.2 µm filter, followed by a 0.1 µm filter (Merck Millipore, Cork, Ireland) and stored at -20 °C. Protein and RNA concentration was analysed pre- and post-depletion and showed a 76.3% reduction in protein and a 65.2% reduction in RNA, as assessed by BCA protein assay and the Agilent 4200 TapeStation (Agilent Technologies, Danta Clara, CA, USA) respectively (Supplementary Fig. 1).

### Pro-inflammatory priming of UCMSCs

Primed UCMSCs were also stimulated with pro-inflammatory cytokines for 48 h when they reached 80% confluence. The pro-inflammatory cytokine cocktail contained: TNF-α (5 ng/ml), IFN-γ (2.5 ng/ml) and IL-1β (2.5 ng/ml) (Peprotech, London, UK) in DMEM F12, 10% EV depleted FBS, 1% P/S.

### Cell Surface Marker Characterisation

UCMSCs (*n* = 4) were characterized using flow cytometry to confirm that cells were of a mesenchymal origin in accordance with The International Society for Cellular Therapy (ISCT) criteria [23]. UCMSCs were harvested at passage 3–5, centrifuged at 500x*g* for 5 min and resuspended in PBS with 2% Bovine Serum Albumin (BSA) (Sigma-Aldrich, Poole, UK). Single-cell suspensions were incubated with Human BD Fc Block™ (BD Biosciences, Wokingham, UK) for 1 h at 4 °C; after which time the receptor block was washed off via centrifugation at 500xg for 5 min. The conjugated monoclonal antibodies against human surface antigens in 2% BSA were added to cell suspensions containing 3 × 10^5^ cells per tube. The cells with antibodies were incubated in the dark at 4 °C for 30 min. The monoclonal antibodies used to identify human surface antigens on MSCs are displayed in Supplementary Table 1. Control samples were stained with IgG controls. Flow cytometry was performed on a FACSCanto II (BD Biosciences, Wokingham, UK) and data were analysed using FlowJo® software (FlowJo LLC v.10.7, US).

### Isolation of EVs using a sucrose cushion

After 48 h incubation, the conditioned media was collected and centrifuged at 300xg for 10 min to pellet cells. The supernatant was collected and spun at 2,000xg for 20 min to remove dead cells, followed by storage at -80 °C until EV isolation.

To isolate the EVs, the conditioned medium underwent differential ultracentrifugation using a 30% sucrose/deuterium oxide (Sigma-Aldrich, Poole, UK) cushion, made up to a density of 1.210 g/cm^3^ [22]. The conditioned media was passed through a 0.22 µm filter (Sarstedt, Leicester, UK), loaded onto a 30% sucrose cushion, and then centrifuged at 100,000xg for 1 h 45 min on an SW28Ti rotor, 25PC Polycarbonate open-top tubes (Scientific Laboratory Supplies, Nottingham, UK) and using an L8-M Ultracentrifuge; *k-*factor 296.8 (Beckman Coulter, High Wycombe, UK). Using a 21G needle and syringe, 3-4 ml of EV suspension was collected at the interface between the sucrose cushion and conditioned media. DPBS was added to this suspension, and it was centrifuged on a Type 70.1Ti fixed angle rotor at 100,000xg for 60 min to pellet pure EVs. All ultracentrifugation experiments were performed at 4 °C and EV pellets were stored at -80 °C. For clarity, this paper uses the term ‘extracellular vesicles’ to describe both exosomes and microvesicles which are smaller than 0.22 µm and float at a density of < 1.2 g/ml, and hence represent small EVs.

### EV Concentration

Protein was extracted from EV samples by lysing samples with cold radioimmunoprecipitation assay (RIPA) buffer (150 mM NaCl, 1.0% IGEPAL®, 0.5% sodium deoxycholate, 0.1% SDS, 50 mM Tris) (Sigma-Aldrich, Poole, UK) for 30 min and sonicating the samples 3 times for 15 s using a Sonomatic Langford sonicator (Agar Scientific, Essex, UK). The protein concentration in the samples was then measured using the Pierce™ BCA Protein Assay Kit (Thermo Fisher Scientific, Waltham, MA, USA) following the manufacturer’s instructions. For Western blot experiments, the samples were normalised to 10 μg of protein for EVs and UCMSCs [16].

### Isolation, culture, and characterisation of PBMCs

Whole blood (~ 6 ml) was obtained from healthy volunteers (*n* = 6) with informed consent in EDTA coated tubes. Blood was obtained from 5 females and 1 male, age range 26–64. Volunteers taking immunosuppressive medication were excluded from the study. Blood was immediately processed to isolate peripheral blood mononuclear cells (PBMCs). PBMCs were separated from blood using density gradient centrifugation over Lymphoprep™ (Stem Cell Technologies, Cambridge, UK). Whole blood was diluted in DPBS at a ratio of 1:1, this solution was then gently layered over Lymphoprep™ and centrifuged at 900xg for 20 min. The blood was separated into layers and the white interphase of PBMCs was collected. PBMCs were resuspended in ice-cold PBS and centrifuged at 500xg for 7 min to remove platelets. The average yield of PBMCs was 1.25 × 10^6^ cells/ml (± 6.02 × 10^5^) (Supplementary Table 4). PBMCs were frozen at -197 °C until needed. Once thawed, PBMCs were cultured in RPMI-1640, 10% FBS, 1% P/S, 10 ng/ml IL-2, at 37 °C, 5% CO_2_.

### Activation of T-cells

Within the mixed PBMC populations, T-cells were activated by adding a humanised CD3 and CD28 agonist (TransAct, Miltenyi Biotec) at a dilution of 1:100. This activation was carried out 48 h before adding them to co-culture experiments. IL-2 (10 ng/ml) was added to the co-culture media of PBMCs. Activation was confirmed by the identification of the surface marker CD25 by flow cytometry.

### Co-culture experiments

A dose–response experiment was first carried out with the co-cultures involving PBMCs (*n* = 3) and control EVs, whereby the EVs were added at 60 μg: 2 × 10^6^, 90 μg: 2 × 10^6^, and 120 μg: 2 × 10^6^ PBMCs cells. The dose–response of PBMCs in co-culture with control EVs was assessed by changes in their protein profile, analysed by flow cytometry.

For functional experiments, 2 × 10^6^ PBMCs (*n* = 6) were co-cultured with 120 μg EVs and 2 × 10^5^ MSCs separately to determine if the EVs or MSCs were able to change PBMC protein expression. 120 μg EVs were obtained from approximately 24.3 × 10^6^ (± 11.4 × 10^6^) UCMSCs. MSCs were added to PBMCs at a ratio of 10:1 (PBMC: MSC) following a protocol by Kay et al. [13] and del Fattore et al. [24]. MSCs were allowed to adhere to plastic for 4 h before the addition of PBMCs to the co-culture. Both control and primed EVs and MSCs were used.

### Surface marker expression and intracellular cytokine staining (ICCS)

After 3 days of co-culture, 2 × 10^5^ PBMCs cells were harvested for immunophenotyping of surface markers or ICCS. Flow cytometry of PBMCs surface markers was carried out using the antibodies listed in Supplementary Table 2. For ICCS, 10 µg/ml Brefeldin A (Sigma-Aldrich, Poole, UK) was added to co-culture for 6 h before harvesting for ICCS. PBMCs were fixed in 1 × Intracellular Fixation Buffer (eBioscience, Waltham, US) for 30 min at 4 °C. Cells were centrifuged at 500xg for 5 min to remove the fixative and the cell pellet was resuspended in 1 × Permeabilization Buffer (eBioscience, Waltham, US). Human Fc Block™ (BD Biosciences, Wokingham, UK) was added at 0.5 mg/ml for 10 min at room temperature before cells were washed in 1 × Permeabilization Buffer. The cell pellet was resuspended in 2% BSA and antibodies were added. All experiments were carried out in triplicate and activated PBMCs alone, acted as a control for the experiments.

### Removal of non-vesicular miRNA

Before RNA isolation from EVs, the EV samples were treated with Proteinase K and RNase A to remove non-vesicular RNAs which may be circulating in the EV suspension or bound to Argonaute2 complex [21, 25, 26]. The EV suspension was incubated in 0.05 mg/ml Proteinase K (Qiagen, Manchester, UK) for 10 min at 37 °C to break down non-vesicular protein complexes. To inhibit Proteinase K activity, 5 mM of phenylmethylsulfonyl fluoride (PMSF) (Sigma-Aldrich, Poole, UK) was added for 10 min at room temperature followed by 5 min incubation at 90 °C. RNase A (Sigma-Aldrich, Poole, UK) was then added to the samples, at a final concentration of 0.5 mg/ml, for 20 min at 37 °C to break down non-vesicular RNAs.

### RNA isolation

Total ribonucleic acid (RNA) was extracted from UCMSCs and EVs using the Qiagen RNeasy® Micro kit in accordance with the manufacturer’s standard instructions (Qiagen, Manchester, UK). RNA was obtained from 3 × 10^5^ UCMSCs, cells were pelleted, washed in PBS, and then lysed using 700 µl QIAzol lysis buffer and stored at -80 °C until RNA extraction. For EV-RNA, 100 µl of EV suspension in PBS was collected from the conditioned media from ~ 15 × 10^6^ cells for RNA sequencing. 700 µl QIAzol lysis buffer was then added to the EV suspension and stored at -80 °C. RNA was eluted in 12 µl of nuclease-free water and stored at -80 °C until quality control steps.

### Quality and quantity of RNA

The quality and quantity assessments of EV-RNA were carried out at the Centre for Genomic Research at the University of Liverpool. The quantity of RNA was determined using the Qubit RNA High Sensitivity™ Assay Kit and the Qubit™ RNA BR Assay Kit (Life Technologies, Waltham, MA, USA) as per the manufacturer’s instructions. The quality and integrity of the RNA were determined by electrophoresis on the Agilent Bioanalyzer 2100 using the RNA 6000 Pico kit (Agilent Technologies, Waldbronn, Germany) following the manufacturer’s instructions. An RNA integrity number (RIN) was generated for the cell samples to assess their quality and a DV_200_ number was generated for the EVs as they do not contain intact ribosomal subunits. This information helped identify the percentage of RNA fragments > 200 nucleotides in the EV samples. All cell samples had a RIN > 8, making them suitable for RNA sequencing.

### Library preparation

The construction of small RNA libraries was carried out at the Centre for Genomic Research at the University of Liverpool with the NEBNext® Multiplex Small RNA Library Prep Set for Illumina (New England BioLabs Inc., Massachusetts, USA) followed by small RNA-Seq analysis on the Illumina NovaSeq SP (Illumina Inc., California, USA). Small RNA transcripts were first converted into cDNA libraries and, following quality and quantity assessments, the final libraries were pooled together in equimolar concentration. The cDNA was Pippin size selected (Sage Science, Beverly, US) with a range set between 130–160 bp. 1 control cell sample, 2 EV samples (control, primed), each with 4 biological replicates was sequenced. Pooled libraries were loaded in the flow cell for cluster formation and sequencing. Samples were sequenced to a depth of 40–100 million reads per sample. Initial file conversion and demultiplexing of sequencing data were carried out at the University of Liverpool. Briefly, the raw Fastq files were trimmed for the presence of Illumina adapter sequences using Cutadapt (version 1.2.1). Reads shorter than 15 bp after trimming were removed and summary statistics were generated using fastq-stats from EAUtils.

### Sequencing data analysis

All further sequencing analysis was carried out at the University of Keele. Raw reads were extracted from the FASTQ files and trimmed of sequencing adapters using Trimmomatic; paired-end reads were merged using the software ‘PEAR’. Phred scores of Q > 30 was taken forward for analysis. The absence of adapter contamination was confirmed using FASTQC. Reads were aligned to Human Genome build (hg38) using miRDEEP2 pipeline. The prepared sequences were filtered and collapsed reads < 18nt were removed to remove uninformative reads as these reads were unlikely to map to miRNA. The trimmed miRNA sequences were mapped to the miRbase sequences to quantify miRNA reads and generate count files. A region 2nt upstream and 5nt downstream of the mature sequence was considered a “hit”. For normalisation and statistical analysis, collapsed reads were passed to DESeq2 (version 1.30.1, Harvard, MA, USA). P-values were created, and a p-value adjusted for multiple testing with the Benjamini–Hochberg procedure was applied. The false discovery rate threshold was set to 10% and an adjusted p-value of < 0.1 was considered statistically significant. A Log_2_ fold change value was calculated by comparing the miRNAs between samples.

### Pathway Enrichment Analysis and Target Prediction

To predict a common pathway of the differentially expressed miRNAs, the web-based analysis tool miRNet 2.0 was used (https://www.mirnet.ca/) [27, 28]. Pathway Enrichment Analysis for predicted gene targets was carried out using the Kyoto Encyclopaedia of Genes and Genomes (KEGG) database and the Gene Ontology (GO): Biological Process database on the differentially expressed miRNAs between the control and primed EVs. The hypergeometric test algorithm was applied to the data. A degree cut-off of 1.0 was selected for optimal visual exploration.

### Statistical analysis

All statistical analysis was performed on GraphPad Prism v.8 (GraphPad Software, San Diego, USA). A Shapiro Wilk test was used to confirm normal distribution. All analysis was carried out using paired Student’s t-tests or 2-way ANOVA. A p-value of ≤ 0.05 was considered significant.

## Results

### Characterisation of UCMSCs after pro-inflammatory priming

**Fig. 1:**
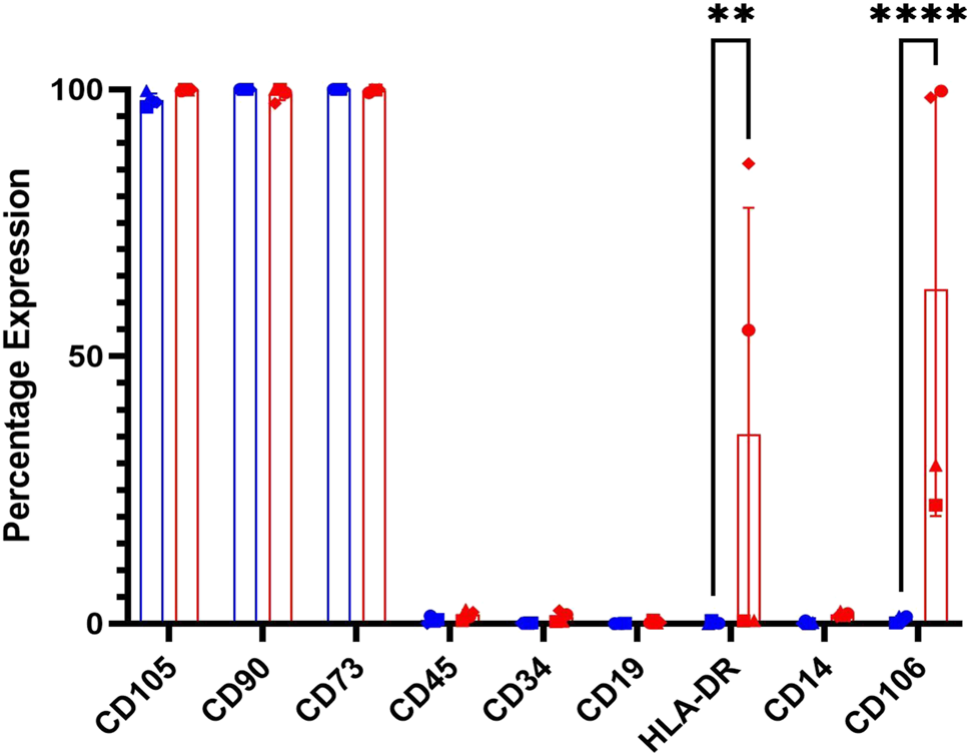
Effect of priming on UCMSCs. *The percentage expression (y-axis) of surface markers (x-axis) from primed/control UCMSCs (n* = 4). There was a statistically higher expression of HLA-DR and CD106 after priming but donor variance was observed, seen by large error bars. The individual donor expression of these markers is thereby denoted using symbols. **p ≤ 0.01, ****p ≤ *0.0001*

Results showed that control UCMSCs at P4-6 were ≥ 95% positive for CD105, CD90, and CD73, and lacked CD45, CD34, CD14, CD19, and HLA-DR (≤ 2%). The primed UCMSCs still displayed the positive (CD105, CD90, CD73) and negative markers (CD45, CD34, CD19) but HLA-DR increased after pro-inflammatory priming (average expression 34.9% ± 39.7 SD)(Fig. 1). This was seen in all donors except for donor 3 in primed UCMSCs. A similar trend was identified for CD106. There was a low level of CD106 (< 2%) detected in all control UCMSCs but its expression increased after pro-inflammatory priming (average expression 57.5% ± 45.3 SD), albeit there was a high donor variability CD106 expression despite the UCMSC samples being donor matched.

### Characterisation of UCMSC-EVs after pro-inflammatory priming

EVs were previously characterised by Hyland et al. [16] to confirm EV isolation. Preparations showed the presence of transmembrane proteins CD9 and CD81 that demonstrate a lipid bilayer structure, cytosolic markers Alix and Hsp70 that show this bilayer contains intracellular content, and lack of GM130 to confirm the isolation of a small EV phenotype. They were also within the size range for EVs as determined using Nanoparticle Tracking Analysis which was confirmed by Transmission Electron Microscopy, revealing a round morphology with a visible phospholipid bilayer. MSC marker CD105 and markers associated with cell adhesion and migration; CD29, CD44, and CD49e, were also present [16]. EV characterisation data are shown in Supplementary Fig. 2. Together, these results show the isolated EV preparations are in accordance with the guidelines for the characterisation of EVs established by the ISEV committee [29].

### Comparison of activated vs non-activated T-cells in the PBMC population

**Fig. 2:**
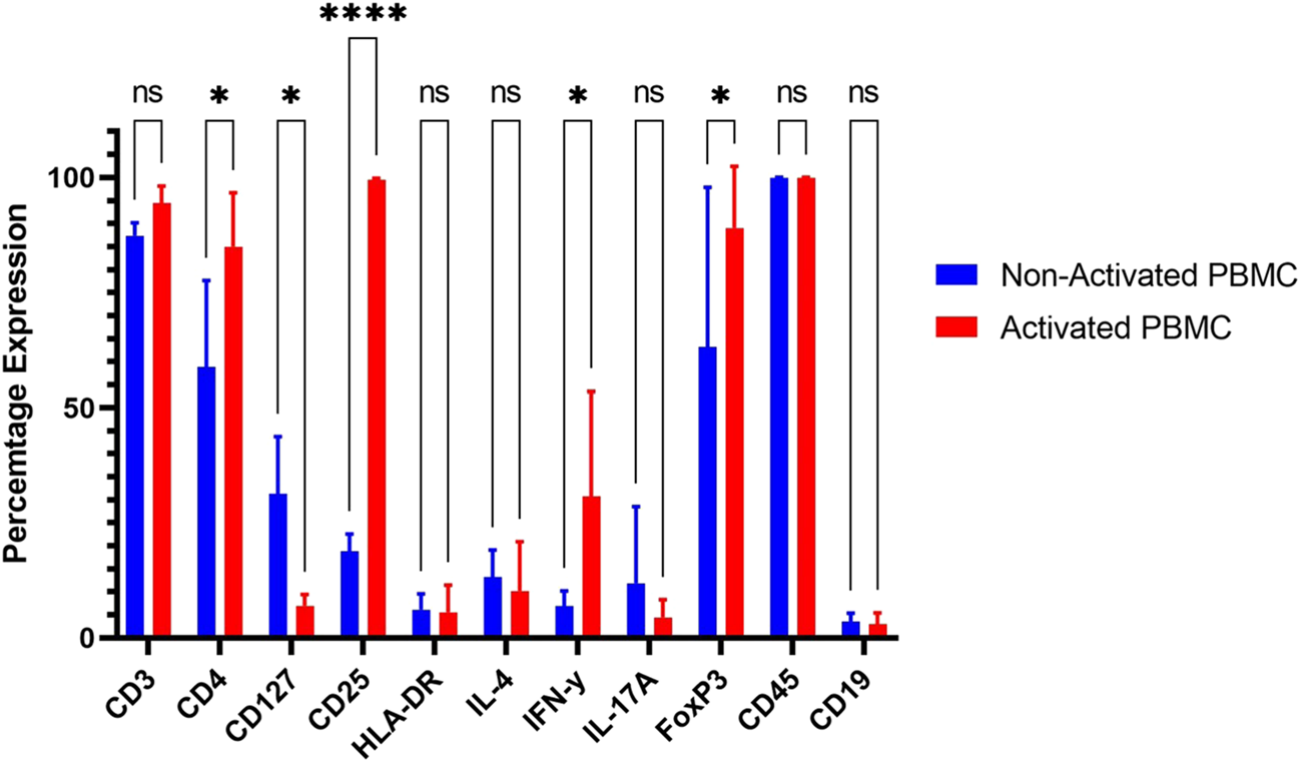
Comparison of Activated vs Non-Activated PBMCs. *PBMCs were activated with CD3/CD28 beads, 48 h before being added to co-cultures for 3 days. Bar chart shows the protein profile of PBMCs (n* = *3) before and after activation. Activated PBMCs (shown in red) had a statistically higher production of CD4 (p* < *0.05), CD25 (p* < *0.0001), IFN-γ (p* < *0.05) and FoxP3 (p* < *0.05), and a lower production of CD127 (p* < *0.05) compared to non-activated PBMCs (shown in blue). Data is shown as the mean* ± *SD*

Following the confirmation that UCMSCs were successfully ‘primed’ and that an EV rich preparation was isolated from these cells, we sought to determine the effect of control and primed UCMSCs, and their derived EVs, on PBMCs. First, the activation of PBMCs was confirmed by evaluating their phenotype using flow cytometry. Non-activated PBMCs contained a 58.85% (± 18.74 SD) expression of CD4 and activated PBMCs had a statistically higher expression at 79.9% (± 13.42 SD) (p < 0.05). Activated PBMCs contained a statistically higher level of CD25 (99.47% ± 0.29 SD), a marker of T-cell activation [30], compared to non-activated PBMCs (18.83% ± 3.76 SD) (p < 0.0001). Further differences were found in the percentage of cells expressing IFN-γ and FoxP3, which was also statistically higher in the activated PBMC group (40.50% ± 25.97 SD) compared to the non-activated PBMC group (6.92% ± 3.29 SD) (p < 0.05). There was a lower percentage of cells with CD127 in the activated PBMCs compared to non-activated PBMCs (7.39% ± 3.37 SD versus 31.33% ± 12.45 SD; *p* < 0.05) (Fig. 2).

### EV dose–response to achieve an anti-inflammatory phenotype in PBMCs

Next, we sought to determine the optimal dosage of EVs to have an anti-inflammatory effect on activated PBMCs. Three different concentrations of control EVs: 60 µg, 90 µg and 120 µg were added to 2 × 10^6^ PBMCs (*n* = 3). The concentrations of 60 µg, 90 µg and 120 µg refer to the total vesicular protein concentration of EVs characterised above (see Characterisation of UCMSC-EVs after pro-inflammatory priming), calculated using a BCA protein assay to normalise EV input [29, 31, 32]. PBMCs were then analysed for their protein marker profile, specifically the expression of pro-inflammatory IFN-γ and IL-17A, and anti-inflammatory IL-4 and FoxP3 using ICCS [4–7].

**Fig. 3:**
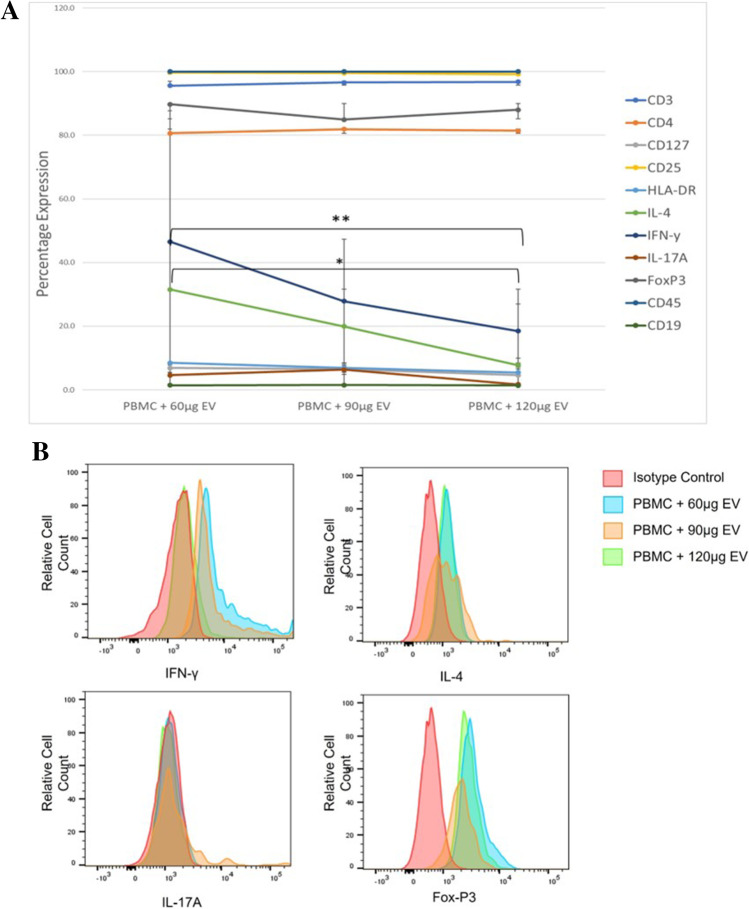
EV dose–response leading to subsequent changes in PBMC phenotype in co-culture. (***A***) *Line graph shows the percentage expression (y-axis) of PBMC (n* = *3) surface markers when co-cultured with EVs of 3 different concentrations: 60 μg, 90 μg, and 120 μg (x-axis). There was a statistically lower expression of IL-4 in the PBMCs that were co-cultured with 120 μg EVs compared to the PBMCs co-cultured with 60 μg EVs (p* < *0.05). IFN-γ had a statistically lower expression in the 120 μg EV co-culture compared to the 60 μg EV co-culture (p* < *0.01)*. (***B***) *Histograms representing the expression of IFN-γ, IL-4, IL-17A and FoxP3 from one donor*

All PBMCs maintained their high expression (> 90%) of CD3, CD25 and CD45 after co-culture. The 60 µg EV co-culture led to a statistically higher expression of IL-4 (31.6% ± 23 SD) in PBMCs compared to the 120 µg EV group (7.73% ± 4.3 SD) (*p* < 0.05) (Fig. 3A), albeit there was a wide range in IL-4 expression (5.13%-46.4%). There was a statistically lower amount of IFN-γ in PBMCs from the 120 µg EV co-culture compared to the 60 µg EV co-culture (18.5% ± 8.5 SD vs 46% ± 41 SD) (p < 0.01)**.** The expression of FoxP3 and IL-17A was consistent in PBMCs across all doses. Therefore, the 120 µg EV concentration was chosen for upcoming co-cultures due to its ability to reduce IFN-γ levels in PBMCs. Representative histograms from one donor are shown in Fig. 3B.

### Immunophenotyping of PBMCs from co-cultures

Following the selection of a 120ug dose of EVs, the immune profile of PBMCs was analysed by flow cytometry after PBMCs were co-cultured with control EVs and MSCs (*n* = 3) and primed EVs and MSCs (*n* = 3). Due to the limited number of PBMCs available for the co-culture experiments, donors 1–3 were used for co-culture experiments with control EVs and MSCs, and donors 4–6 were used for co-culture experiments with primed EVs and MSCs. Activated PBMCs alone acted as a control. PBMCs in all conditions had a > 90.8% expression of CD3, > 95.3% expression of CD25 and a > 98.6% expression of CD45. PBMCs in all conditions were largely negative for CD19 (< 2.7%) indicating that the gated lymphocyte population were primarily of CD3 + origin.

**Fig. 4:**
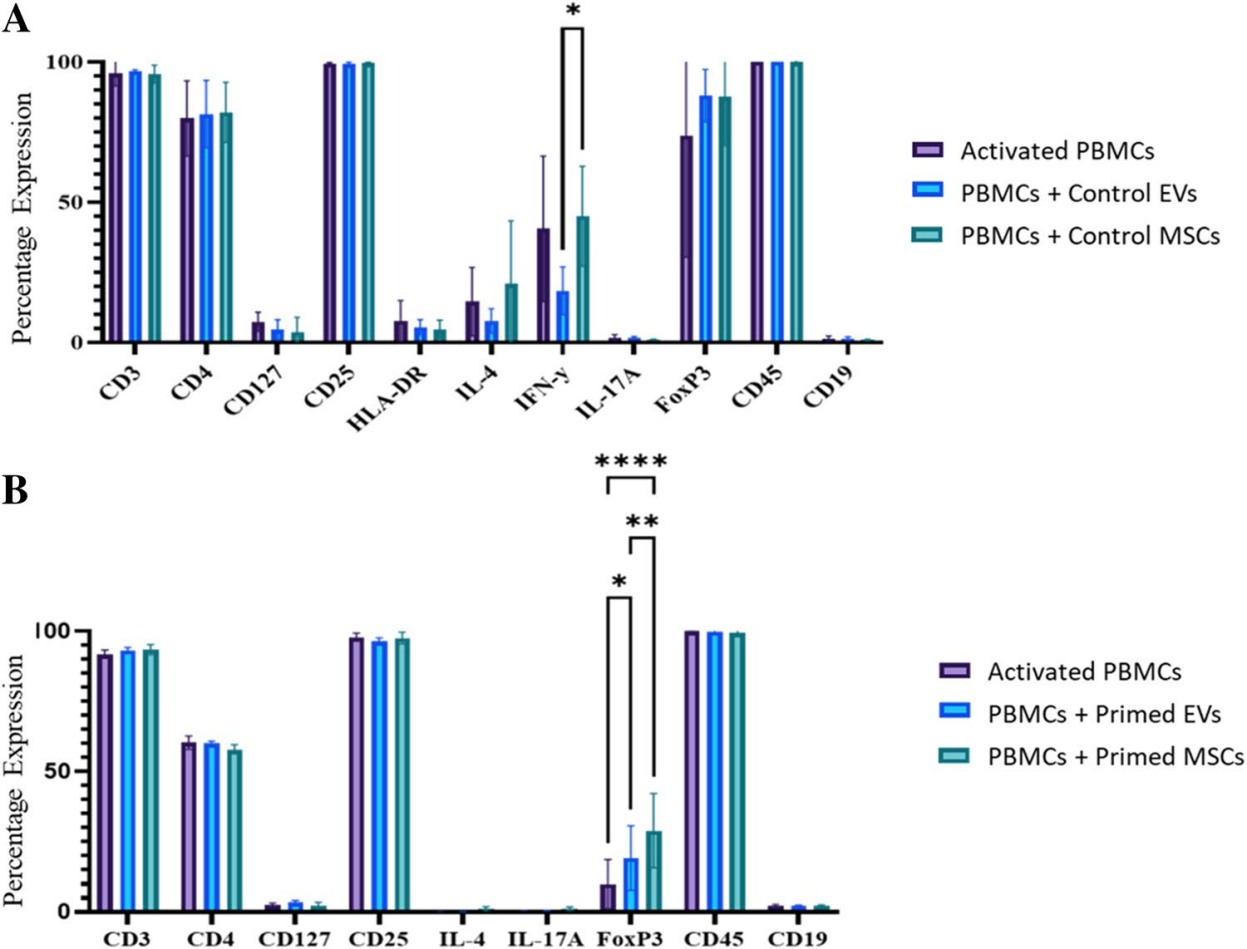
Protein expression of PBMCs after co-culture with control and primed EVs and MSCs. *PBMCs were co-cultured with EVs and MSCs and their protein markers were analysed by flow cytometry. Bar chart displays percentage expression on the y-axis and protein markers on the x-axis.* (***A***) *PBMCs (n = 3) that were co-cultured with control EVs had a statistically lower expression of IFN-γ compared to PBMCs* + *control MSCs (p* < *0.05).* (***B***) *In the PBMC (n* = 3) co-culture with primed EVs and MSCs, there was a statistically higher expression of FoxP3 between primed EVs and MSCs compared to activated PBMCs only (*p < 0.05, ****p < 0.0001). Additionally, PBMCs co-cultured with primed MSCs had a statistically higher expression of FoxP3 compared to PBMCs from the primed EV co-culture (**p < *0.01)*

There was a reduction in the IFN-γ expression in the PBMCs that were co-cultured with control EVs compared to MSCs (*p* < 0.05) (Fig. 4A). There were no further differences between the activated PBMCs, PBMCs + control EVs, and PBMCs + control MSCs. In relation to the PBMCs that were co-cultured with primed EVs and primed MSCs, FoxP3 expression was significantly increased in the PBMCs compared to the activated PBMCs alone (p < 0.05, p < 0.0001). Furthermore, the primed MSCs had a statistically higher FoxP3 expression compared to the primed EVs (*p* < 0.01) (Fig. 4B). There were no further statistical differences between the conditions*.*

**Fig. 5:**
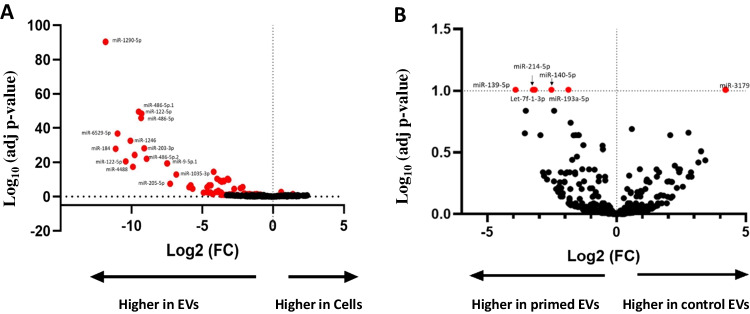
Volcano plot of differentially expressed miRNAs. (***A***) *There was a significantly increased expression of 61 miRNAs (shown in red) from the EVs compared to the cells. Two miRNAs had a higher expression in the cells (miR-127-3p, miR-340-5p) (adj p* < *0.1)* (***B***) *There was a significantly increased expression of 6 miRNAs from the primed EVs compared to the control EVs (adj p* < *0.1). The significance values of the miRNAs are transformed for visual purposes using -log(adj p-value). Values above the horizontal dotted line on the y-axis are statistically significant. Values to the left of the vertical dotted line on the x-axis have a decrease in Log*_*2*_* (FC); values to the right of this line have an increase in Log*_*2*_* (FC). The red dots signify the miRNA’s that reached statistical significance as being differentially expressed*

Having established that primed EVs had the enhanced anti-inflammatory effect in respect to control EVs on activated PBMCs, the next stage was to identify which miRNAs were differentially expressed between the cell and EV samples and the primed and control EVs. An average of 1,126 miRNAs were identified in cell samples and 377 in EVs. In total, 44% of miRNAs expressed in cells were also found in the EVs. 61 miRNAs were differentially expressed between cells and their control EVs (59 had a higher expression in EVs and 2 in cells) (Fig. 5A). This compares to only 6 differentially expressed miRNAs between primed and control EVs, as shown in Fig. 5B. The adjusted p-values were transformed using the negative log for visual representation on the volcano plot. A chart of the differentially expressed miRNAs and their corresponding adjusted p-value is shown in Table 2. All differentially expressed miRNAs between EV conditions had a low normalised read count from the RNA sequencing data (mean 21.4; SD ± 17.2), except for miR-193a-5p, which had an average normalised read count of 290.

**Table 2:** Differentially Expressed miRNAs

	miRNA	Adjusted p-value	Log2 Fold change
Control EVs vs Primed EVs	let-7f-1-3p	9.81E-02	-3.24
	miR-139-5p	9.81E-02	-3.92
	miR-140-5p	9.81E-02	-2.53
	miR-193a-5p	9.81E-02	-1.88
	miR-214-5p	9.81E-02	-3.18
	miR-3179	9.81E-02	4.21
Cells vs Control EVs	miR-1290	4.19E-91	-11.83
	miR-486-5p	3.16E-50	-9.50
	miR-122-5p	3.89E-49	-9.29
	miR-6529-5p	1.71E-37	-10.99
	miR-1246	2.82E-33	-10.09
	miR-203a-3p	7.15E-29	-9.11
	miR-184	1.39E-28	-11.13
	miR-1291	4.55E-25	-9.78
	miR-122-5p	2.12E-21	-10.41
	miR-9-5p	3.62E-20	-7.48

List of differentially expressed miRNAs, their adjusted p-values, and their fold change. There were 5 miRNAs with a higher expression in primed EVs compared to control EVs, and one (miR-3179) with a lower expression. There were 59 miRNAs with a significantly higher expression in EVs compared to cells, and 2 miRNAs with a higher expression in cells. A snapshot of the first ten miRNAs with the lowest adjusted p-value, are shown in the table. A full list of the 61 differentially expressed miRNAs is shown in Supplementary Table 3

The majority of differently expressed miRNAs were of low abundance in the EV/cell samples. The most abundant miRNA expressed across all samples and conditions was miR-21-5p which accounted for 30.3% (± 2.26 SD) of the normalised counts in cell samples, 22.6% in primed EVs and 28.7% in control EVs. In total, the top 10 expressed miRNAs made up 64% of the total miRNA expression in cells, 64% in control EVs, and 62.1% in primed EVs (Supplementary Fig. 3A). A comparison of the normalised gene expression in the most detected miRNAs is shown in Supplementary Fig. 3B. Across all samples and conditions, the top 100 miRNAs accounted for 95–97% of the detectable miRNA sequences, therefore, the remaining 3–5% accounted for low abundance miRNAs.

### Target prediction of differentially expressed miRNAs

**Fig. 6:**
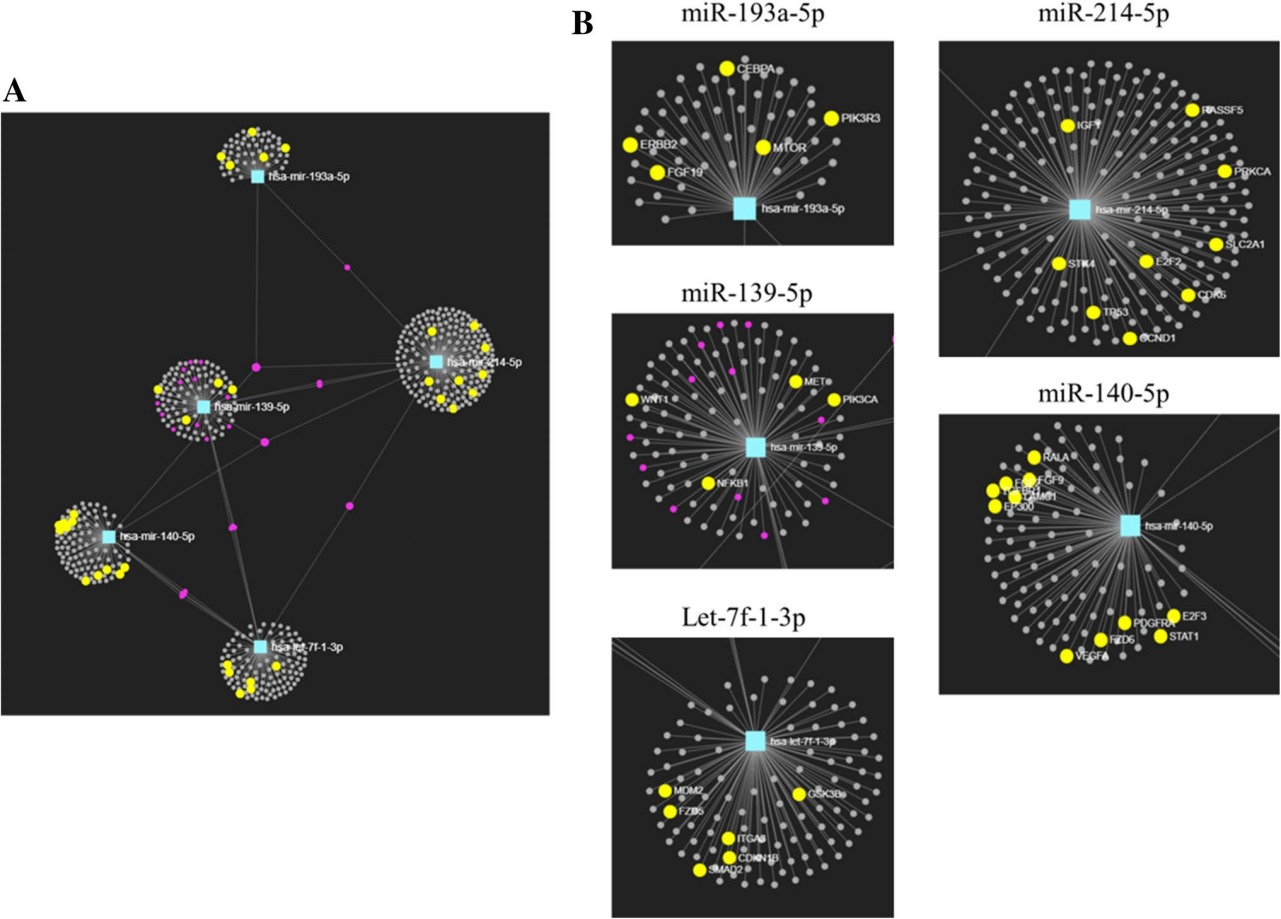
Predicted gene targets in differentially expressed miRNAs using the KEGG database. *Visual representation of the gene targets from miRNet. The miRNAs are identified by a blue box and the gene targets by a pink dot.* (***A***) *Image represents gene targets from five upregulated miRNAs in primed EVs vs the control EVs. Cancer pathways showed the most ‘hits’ in this analysis and these are highlighted in yellow.* (***B***) *Close-up view of the gene targets associated with cancer pathways from the upregulated miRNAs*

Pathway enrichment analysis was carried out on the software ‘miRNet’ using the KEGG database. Analysis was carried out on the five upregulated miRNAs in the primed EVs (let-7f-1-3p, miR-139-5p, mir-140-5p, miR-193a-5p, miR-214-5p). For the 5 upregulated miRNAs in primed EVs, miRNet generated a network comprising of 555 nodes (genes: 550; miRNA: 5) and 565 edges (Fig. 6A), with miR-214-5p showing the most potential gene targets at 188 (Fig. 6B). The KEGG analysis showed that cancer pathways contained the most hits and the most significance overall (p = 2 × 10^–14^), shown in the top 20 pathway enriched pathways (Table 3). To further examine the biological targets of the upregulated miRNAs, the GO: Biological Process database was applied to the dataset [33, 34]. This database was used to support the data from the KEGG database and gain insight into what processes the miRNAs might engage in when inside their target cells. The most significantly enriched pathway from the upregulated primed EV-miRNAs was the ‘negative regulation of cell proliferation’ pathway, this suggest that the primed EVs may be inhibiting cell growth more than the normoxic EVs (Table 3).

**Table 3:** KEGG and GO: Biological Process enrichment analysis

Kyoto Encyclopedia of Genes and Genomes (KEGG) Pathway					Gene Ontology Biological Process (GO:BP) Pathway			
Primed EVs vs Control EVs				Primed EVs vs Control EVs				
Name	Hits	p-value	adj.p-value	Name		Hits	p-value	adj.p-value
Pathways in cancer	41	2.00E-14	2.00E-12	negative regulation of cell proliferation		43	3.16E-07	3.16E-05
Prostate cancer	20	7.73E-12	3.87E-10	intracellular protein transport		50	3.25E-06	1.63E-04
Melanoma	16	7.54E-10	2.51E-08	sensory organ development		35	6.23E-06	1.63E-04
Glioma	14	3.12E-08	7.80E-07	positive regulation of epithelial cell proliferation		15	6.50E-06	1.63E-04
Pancreatic cancer	14	7.01E-08	1.40E-06	epithelial cell differentiation		27	1.29E-05	2.58E-04
Non-small cell lung cancer	11	1.22E-06	2.03E-05	regulation of cell proliferation		73	3.42E-05	5.00E-04
HTLV-I infection	21	4.39E-06	6.27E-05	tube development		34	3.72E-05	5.00E-04
Chronic myeloid leukemia	12	6.58E-06	8.23E-05	positive regulation of transcription from RNA polymerase II promoter		47	4.00E-05	5.00E-04
Focal adhesion	20	1.71E-05	1.90E-04	tube morphogenesis		26	5.29E-05	5.88E-04
Colorectal cancer	9	3.94E-05	3.94E-04	protein targeting		35	7.18E-05	7.18E-04
Bladder cancer	7	4.64E-05	4.22E-04	angiogenesis		29	1.11E-04	8.87E-04
ErbB signaling pathway	11	1.95E-04	1.63E-03	negative regulation of signal transduction		45	1.21E-04	8.87E-04
Cell cycle	13	3.47E-04	2.67E-03	positive regulation of transcription, DNA-dependent		64	1.28E-04	8.87E-04
Small cell lung cancer	10	4.20E-04	3.00E-03	positive regulation of nucleobase-containing compound metabolic process		64	1.28E-04	8.87E-04
Renal cell carcinoma	8	1.04E-03	6.93E-03	MAPK cascade		73	1.33E-04	8.87E-04
Influenza A	11	1.17E-03	7.31E-03	morphogenesis of an epithelium		39	1.92E-04	1.14E-03
Acute myeloid leukemia	7	3.45E-03	2.01E-02	positive regulation of RNA metabolic process		29	1.93E-04	1.14E-03
Endometrial cancer	6	3.95E-03	2.01E-02	intracellular protein kinase cascade		66	2.17E-04	1.15E-03
Epstein-Barr virus infection	9	4.26E-03	2.01E-02	embryonic morphogenesis		58	2.48E-04	1.15E-03
Wnt signaling pathway	12	4.28E-03	2.01E-02	vasculature development		35	2.49E-04	1.15E-03

List of predicted enriched pathways from the KEGG and GO: Biological Process databases. Table shows the upregulated miRNAs in primed EVs vs control EVs. The enriched pathway, hits, p-value, and adjusted p-value are shown in the columns. The term ‘hits’ refers to the number of genes that were direct targets of the miRNAs

## Discussion

The practice of priming MSCs with pro-inflammatory cytokines is a well-researched method to ‘activate’ MSCs to release their pro-inflammatory factors. This paper looked at EVs released from primed and control (non-primed) UCMSCs to see if the EVs too had a heightened immunosuppressive ability after the pro-inflammatory priming of their parental cells.

The primed UCMSCs were first compared to the control UCMSC using flow cytometry to see if they had any differentially expressed markers. Results revealed that HLA-DR had a statistically higher expression in two out of four umbilical cord donors’ primed cells. HLA-DR is an MHC class II surface antigen, known to exist on antigen-presenting cells and plays a role in displaying protein fragments to T-cells, thus facilitating activation [35]. Studies have shown that pro-inflammatory priming, particularly IFN-γ priming, leads to a greater expression of HLA-DR in MSC [36–38] although some papers also report that HLA-DR expression is reduced in MSCs that are grown in pro-inflammatory conditions [14, 39, 40]. This may be explained by the heterogeneity of MSCs, particularly in their response to pro-inflammatory priming. More changes were found in surface marker expression of CD106, also known as vascular cell adhesion molecule 1 (VCAM-1) which also had an increased expression in primed conditions. This is a common finding when MSCs are primed with IFN-γ [39, 41–43] or TNF-α [44, 45]. CD106 is associated with immunomodulation and angiogenesis [46, 47]. Research has previously identified CD106 + MSCs to have greater immunosuppression by reducing pro-inflammatory cytokines IFN-γ and TNF-α, and polarising Th1 cells [42, 46]. This can be explained by CD106 promoting T-cells adhesion, thus bringing T-cells into close proximity of MSCs where they can carry out their immunosuppressive functions [42].

For functional experiments, a dose–response experiment was carried out to establish the optimal concentration of EVs to co-culture with PBMCs. Flow cytometry analysis of PBMC protein expression found that IFN-γ had a statistically lower expression in the 120 µg EV co-culture compared to the 60 µg EV co-culture as shown in Fig. 3A. IFN-γ is produced by Th1 cells and is the main contributor toward inflammation by activating macrophages/monocytes [48], therefore the lower expression of this cytokine in PBMCs points towards an anti-inflammatory effect. This evidence, along with previous research showing that lower EV concentrations aren’t as effective as high concentrations in suppressing pro-inflammatory T-cells [49], led to the decision to use the higher concentration of 120 µg EVs in the co-culture experiments.

The co-culture experiments between the PBMCs and the control EVs/MSCs found that both the control EVs/MSCs were unable to reduce the expression of IFN-γ in activated PBMCs. However, the PBMCs that were co-cultured with control EVs did have a statistically lower expression of IFN-γ when compared to the PBMCs co-cultured with control MSCs, which means that the EVs may be modulating the expression of this pro-inflammatory cytokine to a greater extent than the MSCs. This is different to other studies which have found UCMSCs to be a more potent suppressor of IFN-γ in PBMCs compared to their EVs [50]. When comparing the primed EVs/MSCs to PBMCs alone, FoxP3 was statistically higher in the PBMCs co-cultured with primed EVs and MSCs. No changes were identified in the PBMCs co-cultured with control EVs/MSCs. FoxP3 is a marker of natural Tregs cells [51] and when present on CD4 + CD25 + cells, this population of cells is capable of inhibiting the expression of pro-inflammatory cytokines [52]. This may mean that the pro-inflammatory priming of EVs/MSCs is improving their immunomodulatory abilities by causing them to upregulate FoxP3. Indeed, canine MSC-EV were able to upregulate FoxP3 in PBMCs and the effect of this upregulation was stronger in pro-inflammatory primed EVs compared to control EVs [53].

Once an anti-inflammatory function was established, EVs were then analysed in terms of their miRNA content to identify differentially expressed miRNAs that may contribute to this effect. Of the miRNAs identified through RNA sequencing, 61 were differentially expressed between cells and EVs with 59 miRNAs expressed at higher levels in EVs. This indicates that specific miRNAs were selectively packaged into MSC-EVs, a finding commonly reported in the research [18, 54, 55]. Another objective of this paper was to identify differences in miRNA expression between EVs from control and primed conditions through RNA sequencing. It has been reported that different culture environments such as TNF-α priming [56] can alter the RNA content of EVs. We found that there were 6 differentially expressed miRNAs between primed and control EVs; of which five (let-7f-1-3p, miR-139-5p, miR-140-5p, miR-193a-5p, miR-214-5p) were expressed at higher levels in primed EVs and one (miR-3179) was expressed at a higher level in control EVs.

When the differentially expressed miRNAs are considered individually, their functions in vivo are diverse, and many are understudied regarding immune modulation. MiR-139-5p was upregulated in primed EVs compared to control EVs, which is not surprising as other studies have shown that this miRNA can be upregulated and exclusively shuttled into EVs upon pro-inflammatory signalling [57, 58]. Many studies have focused on the anti-oncogenic properties of miR-139-5p [59, 60]. In terms of miR-139-5p role in inflammation, overexpression of miR-139-5p was found to inhibit MMP9, MMP7, cell proliferation, and pro-inflammatory cytokines (IL-1β, IL-6, TNF-α) in colorectal cancer cell lines [61]. Its mechanism of action involved the suppression of the NF-κB pathway; a signalling pathway that is known to boost the expression of pro-inflammatory cytokines [61, 62]. Other upregulated miRNAs in primed EVs included miR-193a-5p and miR-214-5p. MiR-193a-5p is a commonly detected miRNA in MSCs [63] but few studies have looked at its role in inflammation. One study found that miR-193a-3p reduced intestinal inflammation by targeting the NF-κB pathway [64], but besides this, its function in inflammation remains unknown. MiR-214 is commonly secreted by cancer cells and is associated with cancer progression through targeting of the tumour suppresser gene PTEN [65, 66]. Its anti-inflammatory role was supported by a study of murine kidney disease which showed that the upregulation of miR-214 inhibited TLR4 expression and reduced inflammation [67]. However, few studies have specifically looked at the immunosuppressive role of miR-214-5p in MSCs.

Findings from the pathway enrichment analysis found that primed miRNAs had the most targets to cancer pathways (i.e. this collective set of miRNAs is silencing genes associated with cancer). A direct example of this is miR-139-5p’s ability to silence the cancer-promoting gene BCL-2 [68]. Although, the prevalent connection to cancer pathways may be because cancer is a heavily researched field and studies on miRNAs relating to the immune response are largely understudied [69, 70]. The upregulated miRNAs were also enriched in the ‘negative regulation of cell proliferation’ pathway, this suggests that the primed EVs may be inhibiting cell growth more than the control EVs. Of relevance to this study was the potential identification of miRNAs with anti-inflammatory properties. Enrichment analysis from both databases did not find many ‘hits’ to immune-modulatory functions from the differentially expressed miRNAs. This does not mean that they are not anti-inflammatory, but rather that it is not their main function.

MiR-21-5p had the highest expression in all EV conditions, but, its role in inflammation is more complex [71]. Some studies show that it can stimulate the immune system, particularly eosinophils [69] and T-cells [72]. Other studies show that MSC-EV derived miR-21-5p has anti-inflammatory properties by reducing CCR7 expression on dendritic cells [55]. MiR-21-5p has also been associated with inhibiting Th1 cells, promoting Th2 cells [71], and positively regulating FoxP3 expression in Tregs cells [73]. When considering the miRNA profile of primed EVs and especially the upregulated miRNAs, many connections can be made to anti-inflammatory functions, but the role of these miRNAs in MSCs and MSC-EVs is largely understudied.

Overall, the main aim of this research was to assess the immunosuppressive ability of UCMSCs and UCMSC-EVs on PBMCs. So, the most important question to ask is: are MSCs and EVs immunosuppressive? For the control EVs, they were unable to change the protein profile of PBMCs indicating that they did not make a significant anti-inflammatory change. For the primed EVs, they were able to increase FoxP3 expression in PBMCs; by doing so they have the potential to elicit a biological immunosuppressive change although further testing using a biological assay is required to support this. Nevertheless, these findings indicate that the primed EVs and MSCs are more likely candidates to facilitate immunosuppression compared to the non-primed MSCs and EVs.

This research, however, is subject to several limitations. The first is the availability of the PBMCs donors. Due to the limited number of PBMCs available for the co-culture experiments, different donors were used for co-culture experiments with control EVs and MSCs, and with primed EVs and MSCs. This may explain a discrepancy in FoxP3 expression between control and primed EVs. Also, the dose–response experiments were performed with control EVs only due to the limited numbers of PBMCs available for these experiments.

The second limitation concerns the fact that IFN-γ was not probed for in PBMCs co-cultured with primed EVs and MSC due to issues with antibody availability at the time of the experiments.

## Conclusion

In autoimmune diseases, there is a general imbalance of pro- to anti-inflammatory immune cells and cytokines, so this paper explored the ability of MSCs and EVs to shift the PBMCs to an anti-inflammatory phenotype. Specifically, this paper looked at the protein profile of activated PBMCs after a 3-day co-culture with control and primed MSCs/EVs and the associated miRNAome of primed and control EVs. Results from the co-culture study showed that primed EVs had the most potential to suppress immune responses as they were able to increase FoxP3 expression in PBMCs. Their parent primed MSCs also upregulated FoxP3. No changes were identified in PBMCs that were co-cultured with control MSCs/EVs, therefore, the primed MSC/EVs are most likely to be able to carry out a functional change in PBMCs. Hence, this study sheds light on the influence of MSCs and EVs on PBMCs. It shows that their influence involves the increase of FoxP3 in PBMCs from primed MSCs/EVs. When considering the miRNAome of primed EVs, it does not point towards a group of miRNAs with particularly strong immunomodulatory properties, instead, the pathway analysis showed connections to cancer pathways and promoting cell division, however, there were some differentially expressed miRNAs, such as miR-193a-5p, miR-214-5p and miR-139-5p, with immunomodulatory properties. Few studies have compared the miRNA cargo of proinflammatory primed and control UCMSC-EVs, obtained through RNA sequencing. Overall, this paper data provides valuable data to the understanding of EVs and their miRNA composition, in particular the signalling pathways associated with how EVs counter inflammation. This provides further evidence to supporting the potential use of UCMSC-EVs in treating autoimmune conditions, such as rheumatoid arthritis.

### Supplementary Information

Below is the link to the electronic supplementary material.

Supplementary file1 (PDF 729 KB)

## Data Availability

The datasets generated during and/or analysed during the current study are available from the corresponding author on reasonable request.

## Code Availability (software application or custom code)

N/A.
