# Supplementary material for: Extracellular vesicles derived from umbilical cord mesenchymal stromal cells show enhanced anti-inflammatory properties via upregulation of miRNAs after pro-inflammatory priming
Source: Stem Cell Rev Rep. 2023 Jul 20;19(7):2391–406. doi: 10.1007/s12015-023-10586-2 (PMC10579155; doi:10.1007/s12015-023-10586-2)
Supplement: Supplementary file 1 — Supplementary file1 (PDF 729 KB) [file 12015_2023_10586_MOESM1_ESM.pdf]

Supplementary Figure 1: Depletion of EVs from FBS

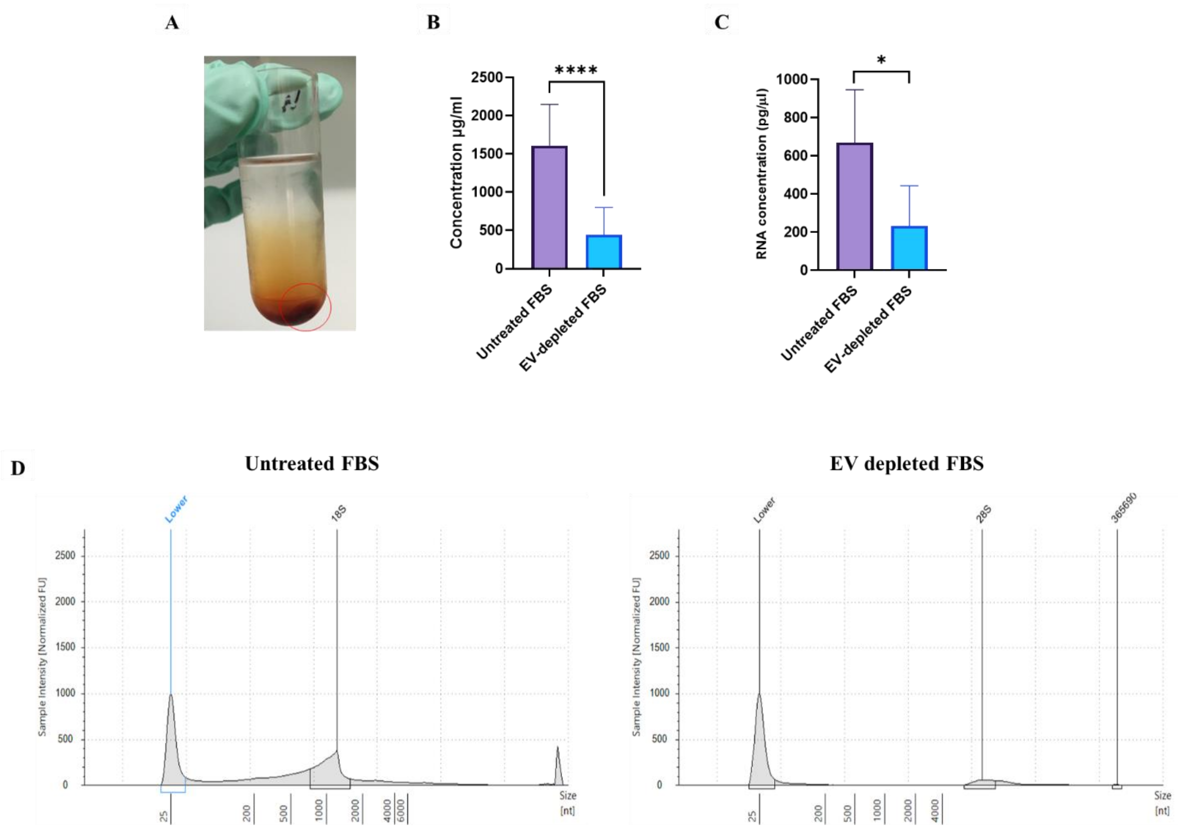

A. Image depicts a visible FBS pellet at the bottom of the tube (circled in red) after an 18-hour ultracentrifugation. The supernatant was collected, avoiding the pellet at the bottom which was likely to contain EVs. B. The protein concentration of the EVs (n=3) isolated from untreated FBS and from the supernatant of ultracentrifuged FBS was measured using a BCA protein assay. The bar chart displays the concentration in µg/ml (y-axis) and the EV-depleted FBS and untreated FBS (x-axis). The protein concentration of the EV-depleted FBS was statistically lower than the untreated FBS ($p<0.0001$) C. Bar chart displays the RNA concentration in pg/µl (y-axis) from EV-depleted FBS EVs (n=3) and the untreated FBS EVs (n=3) (x-axis). The EVs isolated from EV-depleted FBS had a statistically lower RNA concentration compared to the EVs from untreated FBS ($p<0.05$). D. Electropherograms displaying the RNA size profile from EVs derived from untreated FBS and EV depleted FBS. Sample intensity in Normalised Fluorescent Units is shown on the y-axis and nucleotide [nt] size is shown on the x-axis. Electropherograms were used to measure the Dv200 score.

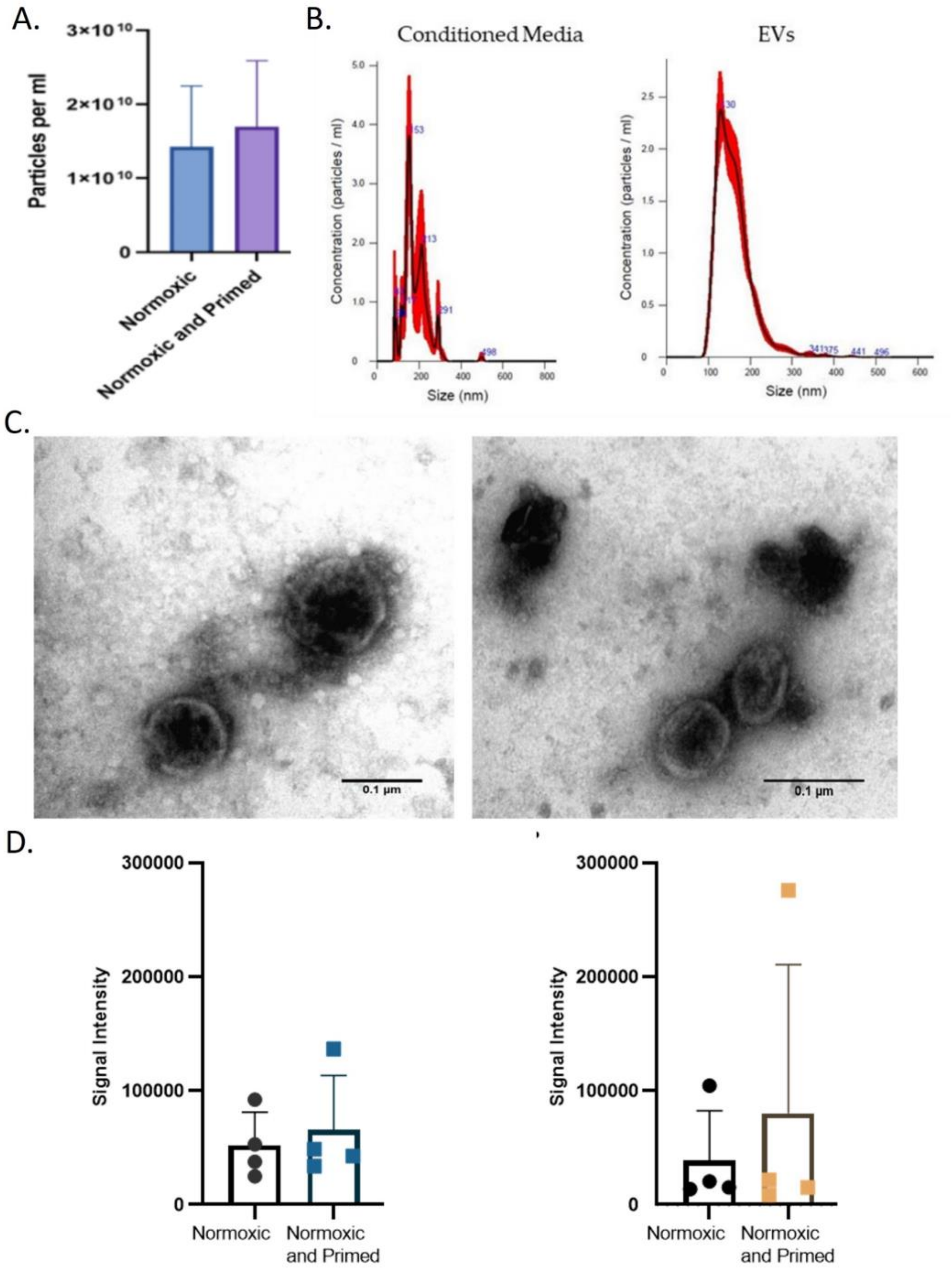

Supplementary Figure 2: EV Characterisation

Image depicts EV characteristics, as represented in Hyland et al. [16]. A. Bar chart representing the particles/ml concentration of the conditioned media (y-axis) from cells grown in different conditions (x-axis). B. Size profile of particles from conditioned media and particles from isolated EVs. The conditioned media has a more diverse size profile seen by many small peaks compared to the EVs which show a more uniform size profile, seen by one clear peak. C. Images of EVs in normoxia taken using TEM. EVs have a round morphology, size of ~100 nm and a visible bilayer membrane. Scale bars = 0.1 µm. D. Histogram showing the expression of tetraspanin markers CD9 (left) and CD81 (right), in EVs (n=4) from each condition detected using a europium-based immunoassay. Signal intensity is displayed on the y-axis and the EV conditions on the x-axis. All figures shown were calculated after subtracting the isotype control. Error bars indicate mean ±SD.

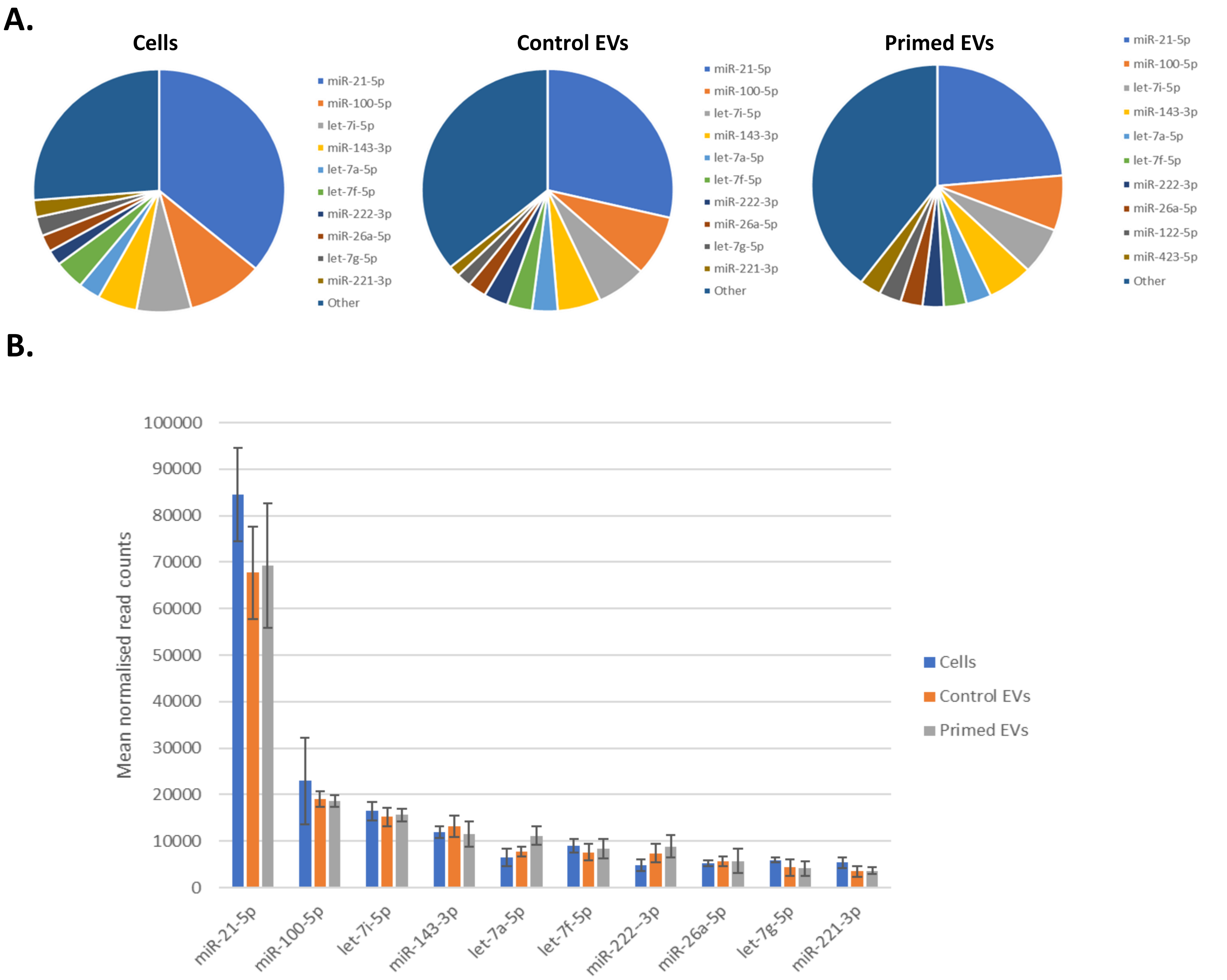

Supplementary Figure 3: Profile of commonly expressed miRNAs in cells and EVs

A. Pie charts displaying the 10 most expressed miRNAs in cells, control EVs and primed EVs. **B.** Bar chart comparing the expression of some of the most expressed miRNAs across samples and conditions. The miRNAs are presented on the x-axis and the mean normalised read count on the y-axis. There were no statistical differences between the samples for the miRNAs presented here.

Supplementary Table 1: List of antibodies used to characterise UCMSCs

All antibodies were directly conjugated to a fluorochrome and used in a working concentration of 5μg/ml. All antibodies were purchased from BD Biosciences, Wokingham, UK.

Antibody	Conjugated Fluorochrome	Dilution	Isotype Control
CD105	APC	1:20	APC Mouse IgG1
CD90	PE-CF594	1:20	PE-CF594 Mouse IgG1
CD73	BV421	1:20	BV421 Mouse IgG1
CD14	PerCP-Cy5.5	1:20	PerCP-Cy5.5 Mouse IgG2b
CD45	PE-CF594	1:20	PE-CF594 Mouse IgG1
CD34	APC	1:5	APC Mouse IgG1
CD19	BV421	1:20	BV421 Mouse IgG1
CD106	APC	1:2.5	APC Mouse IgG1
CD146	PE-CF594	1:11	PE-CF594 Mouse IgG1
HLA-DR	APC	1:5	APC Mouse IgG2b
APC Mouse IgG1	APC	1:5	
PE-CF594 Mouse IgG1	PE-CF594	1:50	
BV421 Mouse IgG1	BV421	1:100	
PerCP-Cy5.5 Mouse IgG2b	PerCP-Cy5.5	1:5	
APC Mouse IgG2b	APC	1:5	

Supplementary Table 2: Antibodies used in the characterisation of PBMCs

All antibodies were purchased from BD Biosciences, Wokingham, UK.

Antibody	Conjugated Fluorochrome	Dilution	Isotype control
CD3	APC	1:20	APC Mouse IgG1
CD4	PE	1:20	PE Mouse IgG1
CD127	BV421	1:50	BV421 Mouse IgG1
CD25	APC	1:50	APC Mouse IgG1
CD45	PE	1:20	PE Mouse IgG1
CD19	BV421	1:100	BV421 Mouse IgG1
HLA-DR	APC	1:5	APC Mouse IgG2b
IL-4	PE	1:20	PE Mouse IgG1
IL-17A	BV421	1:50	BV421 Mouse IgG1
IFN-y	PerCP-Cy5.5	1:20	PerCP-Cy5.5 Mouse IgG2b
FoxP3	PE	1:20	PE Mouse IgG1

Supplementary Table 3: List of differentially expressed miRNAs between cells and EVs

Table displays a full list of the 61 differentially expressed miRNAs between control cells and control EVs along with the Log2(FC) and the adjusted p-value. 59 miRNAs had a higher expression in EVs, and 2 miRNAs (miR-127-3p and miR340-5p) had a higher expression in cells.

	Log2 (FC)	Adjusted p-value		Log2 (FC)	Adjusted p-value
hsa-miR-1290	-11.83	4.19E-91	hsa-miR-142-3p	-4.41	0.003162
hsa-miR-486-5p	-9.50	3.16E-50	hsa-miR-200b-3p	-3.67	0.003303
hsa-miR-122-5p	-9.29	3.89E-49	hsa-miR-6131	-4.90	0.004151
hsa-miR-6529-5p	-10.99	1.71E-37	hsa-miR-323a-5p	-4.53	0.004944
hsa-miR-1246	-10.09	2.82E-33	hsa-let-7c-5p	-2.04	0.015102
hsa-miR-203a-3p	-9.11	7.15E-29	hsa-miR-543	-1.99	0.01641
hsa-miR-184	-11.13	1.39E-28	hsa-miR-92b-3p	-1.65	0.025211
hsa-miR-1291	-9.78	4.55E-25	hsa-miR-3158-3p	-2.87	0.029505
hsa-miR-122-5p	-10.41	2.12E-21	hsa-miR-5100	-3.64	0.029505
hsa-miR-9-5p	-7.48	3.62E-20	hsa-miR-340-5p	1.39	0.031254
hsa-miR-4488	-9.91	3.40E-18	hsa-miR-6826-3p	-4.26	0.033039
hsa-miR-126-3p	-4.19	2.82E-15	hsa-miR-1307-3p	-1.28	0.034358
hsa-miR-10395-3p	-6.84	1.13E-13	hsa-miR-432-5p	-1.80	0.034705
hsa-miR-423-5p	-3.95	3.33E-11	hsa-miR-760	-3.07	0.045366
hsa-miR-320b	-3.14	1.37E-10	hsa-miR-148b-5p	-3.07	0.046511
hsa-miR-1-3p	-3.74	4.16E-10	hsa-miR-485-5p	-2.02	0.046511
hsa-miR-320c	-3.48	1.01E-09	hsa-miR-151a-3p	-0.90	0.050443
hsa-miR-205-5p	-7.27	3.12E-08	hsa-miR-409-3p	-1.49	0.055556
hsa-miR-320d	-4.52	2.17E-07	hsa-miR-615-3p	-1.95	0.0588
hsa-miR-215-5p	-4.39	2.31E-07	hsa-miR-423-3p	-0.99	0.063741
hsa-miR-4448	-5.82	2.31E-07	hsa-miR-128-3p	-0.99	0.070354
hsa-let-7b-5p	-2.14	2.00E-06	hsa-miR-200c-3p	-2.85	0.07534
hsa-miR-142-5p	-5.92	2.97E-06	hsa-miR-92a-3p	-0.85	0.084618
hsa-miR-192-5p	-2.29	1.17E-05	hsa-miR-3605-5p	-3.29	0.095663
hsa-miR-4671-5p	-5.68	2.21E-05	hsa-miR-363-3p	-3.64	0.095663
hsa-miR-320a-3p	-2.74	2.75E-05	hsa-miR-92b-5p	-2.50	0.095663
hsa-miR-193b-5p	-3.87	0.000365	hsa-miR-99a-5p	-1.16	0.095663
hsa-miR-320e	-3.86	0.001598	hsa-miR-4680-3p	-3.45	0.096476
hsa-miR-127-3p	0.58	0.001676	hsa-miR-744-5p	-0.96	0.097327
hsa-miR-342-5p	-4.01	0.001676	hsa-miR-1228-5p	-3.29	0.097544
hsa-miR-3179	-4.65	0.002396			

Supplementary Table 4: PBMC yield

List of the PBMC yield in cells/ml (n=6).

	PBMCs (cells/ml)
**Donor 1**	1.57×10^6
**Donor 2**	1.51×10^6
**Donor 3**	1.05×10^6
**Donor 4**	0.42×10^6
**Donor 5**	0.86×10^6
**Donor 6**	2.11×10^6
